# The Healthy Eating Agenda in Australia. Is Salt a Priority for Manufacturers?

**DOI:** 10.3390/nu9080881

**Published:** 2017-08-15

**Authors:** Rebecca Lindberg, Tyler Nichols, Chrystal Yam

**Affiliations:** The Australian Health Policy Collaboration, College of Health and Biomedicine, Victoria University, 300 Queen St, Melbourne, VIC 3000, Australia; tyler.nichols1@vu.edu.au (T.N.); chrystalyam@yahoo.com.au (C.Y.)

**Keywords:** salt, food policy, food reformulation, food industry

## Abstract

Many nation states have endorsed and acted on the World Health Organization’s target of a 30% reduction in global salt consumption by 2025. In Australia, new government-led voluntary measures were initiated in 2009, consisting of public–private partnerships, front-of-pack labelling, and food reformulation targets (which include reduced salt). How Australia’s private sector has responded to this healthy eating agenda has been investigated in a limited way, particularly with regards to manufacturers which produce processed foods considered significant sources of sodium. In this study we asked: have Australia’s largest food manufacturers made “…positive (nutrition) changes to their product portfolios” as disclosed in their public policies, priorities, and communications? And, is salt reduction a priority for processed food manufacturers? A systematic search and critical content-analysis of grey literature published by food manufacturers was conducted. The results suggest half of the sample publically describe some salt reduction activities but the scale and efficacy of these changes is unclear from the available literature. The Australian Government’s Healthy Food Partnership could capitalise on current documented activities in salt reduction, and implement a more comprehensive healthy eating agenda moving forward. In light of the increasing rates of hypertension, population salt consumption and diet-related disease, more could be done.

## 1. Introduction

Non-communicable diseases (NCDs), including cardiovascular and cerebrovascular disease, kill more people each year than all other causes combined [1]. The World Health Organization’s (WHO) Global Action Plan for the prevention and control of NCDs [1] contains nine voluntary global prevention and reduction targets [1]. Two of the targets include a 30% reduction in salt intake and 25% reduction in raised blood pressure. These were set because of the known association between these risk factors and cardiovascular and cerebrovascular diseases, and other NCDs [2]. The Global Action Plan explicitly encourages collaborative partnerships between government, civil society and the private sector to achieve the targets by the year 2025.

Unfortunately, Australians are eating more salt than ever [3]. Almost one quarter of adults (23.1%) have high blood pressure and cardiovascular disease is Australia’s most expensive disease group [3]. Adults consume an average of 9 g of salt per day—well above the WHO recommended daily intake of 5 g [4]. It is estimated that 75–80% of salt consumed is via “hidden salt” in processed foods [5]. These foods (Table 1) [6] include baked goods, cereal based products, processed meat, soup and sauces and may be prepared and consumed at home and/or sourced from quick service outlets and restaurants.

Reducing salt content in processed products is one of the most cost-effective preventative population health interventions [7]. Even modest reductions across the supply result in a substantial decrease in population intake and subsequently deliver health outcomes [8,9]. However, influencing and implementing food reformulation strategies is not without difficulty [5,10]. Consumer acceptance of reformulated products, manufacturing limitations, food safety, and quality and shelf-life trade-offs are all potential issues [10,11,12]. Despite these challenges, salt levels can be reduced by approximately 40% in breads and 70% in processed meat products without affecting consumer acceptability [12]. In fact most challenges can be mitigated or resolved and hence food reformulation is increasingly being supported by governments, advocated by civil society and considered and implemented by food industry.

### 1.1. International Policy Context

Influenced by the Global Action Plan and mounting evidence and advocacy on the need for changes in the food environment, over 80 countries have adopted national salt reduction strategies [13]. Of these strategies, 71 either include or plan to include, programs that engage food industry to achieve salt reduction at a population level [13]. In terms of high and middle-income countries, the United Kingdom (UK) was one of the first to adopt a national salt reduction strategy predating the Global Action Plan. Their 2003 strategy included voluntary reduction targets applied to more than 80 food categories, public awareness campaigns and mandatory labelling of high salt foods [13]. Despite the voluntary nature food manufacturers did, and continue to participate, with several publically disclosing their commitment and achievements in salt reduction [14,15,16,17]. The current targets are detailed within the Department of Health’s “Public Health Responsibility Deal” [18]. Signatories to the “Deal” update their progress towards the targets on an online platform administered by Government [18]. Overall, in the last decade the UK has achieved a 15% reduction in population salt consumption [13]. Maintaining this progress requires ongoing monitoring, accountability and Governmental leadership [19].

Argentina and South Africa have displayed significant leadership by implementing mandatory maximum salt levels on a range of staple foods [13]. Food manufacturers have time frames in which they are required to comply, or face sanctions. Additionally, legislation in Argentina is applicable to the hospitality sector by setting maximum salt content in meals supplied in quick service and restaurants [13]. Mandatory targets may seem heavy-handed given the success of the voluntary system in the UK, but there is some evidence that even within the British food industry mandatory targets are preferable as they level the playing field [20,21].

### 1.2. Australian Policy Context

Australia has an international commitment to address noncommunicable diseases in line with the Global Action Plan. In Australia, a national collaboration of public health experts has adapted the Global Action Plan and the associated targets for the Australian setting [3]. This equates to reducing the average population salt intake to 6 g, and the proportion of the population affected by high blood pressure to 16.1%, by the year 2025 [3]. From 2009 to 2014 the Australian Food and Health Dialogue (FHD) was in operation as a public–private partnership to improve healthy eating. Its stated goals were admirable but the mechanism for implementation and accountability was criticised as weak [22,23,24].

Sodium (as a proxy for salt) was one of several reformulation action areas considered under the FHD [24]. Only 12 out of a possible 137 reformulation action areas had voluntary targets set for them over the duration of the FHD, nine of these regarding sodium [24]. While this indicates slow and disappointing progress in setting targets, it suggests that sodium reduction was a priority under the FHD. An evaluation of nine of the product groups (summarised in Table 2 [25]) shows good progress on food reformulation and compliance with the maximum sodium levels. However, these voluntary targets have been criticised as unambitious and likely to result in only minor effects to population intake [23].

Due to a change in national governments, the FHD was inactive for a 1–2 years period [24] and re-emerged as the Healthy Food Partnership (The Partnership) in November 2015. The Partnership continues to support the voluntary front-of-pack labelling Health Star Rating scheme [24] and in 2016 appointed a food reformulation working group to review the existing targets and explore expansion of the voluntary targets (including sodium) to other food categories [26]. The Partnership website [26] states:

The Australian Government, food industry bodies and public health groups have agreed to cooperatively tackle obesity, encourage healthy eating and empower food manufacturers to make positive changes to their product portfolios.

### 1.3. Study Aim and Research Questions

At this mid-point between setting the Global Action Plan and the year 2025, it is timely to assess the adequacy of the Australian government’s action on salt reduction and NCDs to date. Comprehensive accountability for nutrition action typically involves gathering information, monitoring and measuring financial or institutional performance against the mandatory or voluntary standards, and utilising this intelligence to improve performance [27]. Gathering information for this purpose can include a range of methods and analysis, including an investigation of food and beverage manufacturers’ policies, practices and disclosure of their contributions to improving nutrition [28].

In this study we sought to contribute to accountability and answer the research questions:

- Have Australia’s largest food manufacturers made “…positive (nutrition) changes to their product portfolios” as disclosed in their public policies, priorities, and communications?
- And, is salt reduction a priority for processed food manufacturers?

## 2. Materials and Methods

In order to answer the research questions, we critically appraised Australia’s largest food manufacturer’s public priorities and reported actions in relation to healthy eating and salt reduction in processed foods. A systematic search of grey literature published by food manufacturers was designed and conducted to appraise stated priorities and achievements. Grey literature (such website content, policies, media releases) from 2010 to 2017 was sought to coincide with the Global Action Plan, the FHD and the Partnership.

### 2.1. Search Strategy

The largest food manufacturers (as defined by the company’s net profit in the 2015–2016 financial year) were identified via the Ibisworld “Australia’s Top 100 Food and Drink Manufacturers” publication [29]. The list was appraised by the first and second authors to include Australian manufacturers of relevant products (i.e., processed foods described in Table 1 and Table 2). Manufacturers that exclusively produced or processed alcoholic beverages, fresh meat/abattoirs, or fruit and vegetables, were excluded. Importers without Australian production capacity were also excluded as we prioritised Australian-made products to assess the Australian Government-led healthy eating agenda.

Each inclusion/exclusion decision was recorded. Discrepancies were discussed between the first two authors and resolved, recording the outcome. For example, one author may have located a manufacturer that produced well-known products in the Australian market, whereas the second author may have correctly identified that the company imports these goods. The final included list was checked by the third author.

After initial screening, the websites of included manufacturers were further investigated and searched for descriptions of the company’s commitment or activities relevant to healthy eating and salt or sodium reduction. The search occurred February–May 2017. Where possible, within website search boxes were used, applying the following terms:

- Health
- Nutrition
- Salt
- Sodium
- Reformulation

If the website did not have a search box function, the first and second authors manually reviewed the website home page and sub-pages including nutrition sections, corporate social responsibility sections, annual reports and media sections. The title of media releases, statements on the website, nutrition policies, corporate social responsibility plans or any other content that was returned in the search was reviewed. Based on the titles relevant grey literature were saved. The “about us” (or similar) section of each website was also downloaded, as was each manufacturers most recent annual report (2016) and/or corporate responsibility report (2016 or 2017). Manufacturers that produced several brands were noted, and the branded website was also searched. For international companies, the Australian version of their site was searched.

Included manufacturers were contacted via email/online enquiry forms, to provide them with the opportunity to add additional published material. All grey literature were added to EndNote and used for content analysis.

### 2.2. Synthesis and Analysis

The included grey literature were read in full by the first author and the content for each manufacturer was manually inductively analysed, colour coding the literature. Evidence of food reformulation, participation in voluntary measures including the Health Star Rating and/or the Partnership, introduction of new healthier product lines and targets for reduced sodium content were recorded. Nutrition priorities such as action or policies that restrict marketing to children, support reductions in energy content, serving sizes, sugar and saturated/trans-fats and/or increased fibre, new healthier product lines, manufacturing of healthy nutritious foods and consumer information were also recorded. Other priorities were noted including local production, philanthropy, and environment as these themes often overlapped and were communicated alongside manufacturer’s nutrition priorities.

The results were summarised in a table and audited by the second and third authors to triangulate the findings and resolve any discrepancies. The authors then focussed on the manufacturers where salt/sodium was included in their public communications and attempted to document the extent of the commitment, progress made and transparency in goals. Data immersion occurred by reading and re-reading the content, discussing emergent ideas and subsequently co-analysing and co-coding the data to agree on themes relevant to the research questions.

## 3. Results and Discussion

Thirty-three of Australia’s 100 largest manufacturers make product lines of relevance to salt-reduction and therefore, were included in the study (Table 3). One-hundred and forty three grey literature were reviewed to extract data on manufacturers, including website content, media releases, policies, annual reports and emails from company representatives.

This study found that over half (*n* = 17) of the 33 manufactures disclosed that they were reducing salt in at least some of their food products (see Table 3). All of the manufacturers provided some evidence of nutrition and healthy eating as a part of company’s policies, protocols and priorities (see Table 4). Interestingly the content included in the inductive analysis suggested that most manufacturers also reported the environment and sustainability as a part of their priorities and this emergent theme will be briefly discussed further below (Section 3.3). Other priorities for manufacturers include quality control and food safety, community development and philanthropy, gender diversity and workplace culture, regulatory compliance, commitment to Australian made and local jobs, and expansion into Asia and abroad.

The main themes relevant to the research questions will be expanded on below and potential reasons for these findings will be discussed. The implications for policy and practice will also be considered.

### 3.1. Salt Reduction in Manufacturers’ Product Portfolios

Manufacturer’s policies and priorities on salt reduction could be categorized in three main ways: those with “no evidence”, those with “some evidence” and those with “considerable evidence”.

This study found that 16 out of 33 companies that produce processed foods in the Australian market that are considered significant sources of sodium, provide no evidence or documentation of reducing salt in their products (see Table 3). These manufacturers may be reducing salt and not disclose this, or may have no commitment to reformulation or salt reduction. Cheese, processed meat products such as hamburgers, pies and chicken nuggets, pasta and pasta sauces, crackers and snack foods had voluntary sodium reformulation targets during the FHD from 2010 to 2014 and the adoption and/or maintenance of these targets is not evident among these manufacturers’ publically available documents. The Healthy Food Partnership has not yet endorsed the continuation or expansion of the targets although this was due February 2017 [30]. Technical, financial or consumer-based barriers may be real or perceived challenges to reformulation by these manufacturers [5,10].

Of the 17 manufacturers that disclosed some action on salt-reduction, by investigating the literature to identify the extent of the commitment, progress made and transparency in goals, it appears that most manufacturers had broad aspirations or achievements for their products (Table 3). For example Coca-Cola Amatil report they “…reduced salt and sugar in key tomato products helping Australians eat healthier” [31]. Similar statements about tonnes of salt removed from the food supply, “reduced salt” product lines, and participation in front-of-pack labelling schemes imply that companies are aware of the high levels of population salt intake, the government-led agenda and/or the consumer demand for reduced salt and are therefore making positive changes to their portfolio. The scale and efficacy of these changes is unclear from the publically available literature.

Manufacturers such as Mondelez, Nestle and Unilever are among the largest multinational food companies in the world and they appear to be, in comparison to the rest of the sample, more transparent and provide considerable documentation of their achievements and aspirations for food reformulation. Both Mondelez and Unilever have corporate responsibility progress reports that describe their salt-reduction (and other) priorities, progress against targets and partnerships to help achieve the targets [32,33]. Mondelez’s 2015 report [32] notes the target of a 10% reduction across all product lines by 2020 and significant progress in food-reformulation in Latin American Oreo biscuits and Ritz crackers in the UK. Interestingly these are the regions where the company also participates in the Pan-American Sodium Consortium and the UK Responsibility Deal, suggesting public–private partnerships can trigger progress on healthier products. Oreo and Ritz biscuits are international products and those sold in Latin America and the UK respectively are lower in salt than in Australia. Mondelez states these reductions in these markets is a significant achievement [32]. This implies the reductions have been achieved without reducing consumer acceptability.

Nestle and Unilever state that they have adopted the WHO recommended upper intake level of 5 g salt per day and formulated their products to help consumers to not exceed this level [33,34]. The Unilever website includes a table of their nutrition criteria for maximum level of salt in a range of product groups, a position statement on salt reduction and progress, by country, towards their 2020 target. In Australia 68% of products sold by Unilever meet levels to enable 5 g/daily [33]. These companies appear to undertake the research and development, partnership, and monitoring that enables reporting and food reformulation. There is evidence of the leadership from senior levels within the company of food reformulation and public accountability as demonstrated in the stated responsibilities expected of the Board of Directors [35]. These companies may offer a model for other manufacturers.

The results suggest that of 33 Australian food manufacturers included in this study approximately half document salt-reduction efforts in their publically available literature. Participation in government-led measures and/or salt reduction was outlined in greatest detail by the multi-national food companies. The efficacy and scale of the “positive (nutrition) changes” is unclear from the content included in this study and regular independent monitoring of food reformulation activities in Australia would be preferable.

### 3.2. Salt and Other Nutrition Priorities

There was documentation of nutrition activities and policies for all of the included manufacturers (see Table 4) but the scope, validity of claims and effect of these activities is unclear from the available literature. Several included manufacturers make nutritious foods consistent with national dietary guidelines including dairy, poultry and cereals. It was common to find grey literature that educated the consumer about the nutritional benefits of products and/or how to interpret food labels using nutrition information panels, daily intake guides and the Health Star Rating system. George Weston Foods, for example, reports in their corporate responsibility document that they are a member of the GoScan “app” program. On their Tip Top (bread manufacturer) website [36], the frequently asked questions section provides information on ingredients, the benefits of fibre and the function of salt as an ingredient in bread. Several manufacturers employed dietitians to blog, provide recipes and information.

Some manufacturers (see Table 4) also described reformulating foods to reduce saturated and trans fats, sugar, energy and increase fibre and protein. Snack food companies also disclosed their compliance with responsible marketing to children initiatives [37,38] and several mentioned compliance with school canteen guidelines [39,40]. In comparison to other risk-associated macro or micro-nutrients, salt received no more or less attention.

It is commonly understood that websites and public documents are designed to create brand loyalty and provide consumers and investors with information. Therefore enabling consumers to “…access trusted product information” [41] to benefit their nutrition and health, is not out of the ordinary. However, it also reinforces the common expectation of individual responsibility for dietary choices and behavior, and could be considered as a way to distance corporations from responsibility for the nutrition of their food products.

In answer the first research question, it is unclear from industry’s public priorities and policies if “…positive (nutrition) changes to… product portfolios” are comprehensively occurring in the Australian food supply, despite the reported nutrition activities. Therefore, it is also uncertain if the Partnership is achieving its goals after 18 months of work. In answer to the second research question, salt reduction appears to be one of several nutrition and healthy-eating priorities for most processed food manufacturers. It is encouraging that half of the sample report some activity to reduce salt and now more can be done to comprehensively reduce population sodium consumption.

### 3.3. Manufacturers Responsibilities

The inductive analysis suggests that some, mainly the large and multinational companies disclose three, four or five “responsibilities” that they implement and aspire to [31,40,41]. Community and philanthropy is typically one, the environment is another and healthier and higher quality products for consumers is often the third. These triple-bottom line principles appear well enshrined in the corporate culture of large multinational food manufacturers, although evidence of food companies not behaving responsibly also exists [42].

The environment was a common publically articulated priority by manufacturers in this sample. The Australian Packaging Covenant [43], and compliance with waste, water and pollution standards were often reported. So called “greenwashing” has been identified in food [44] and other companies but innovation to benefit the environment by the private sector also occurs. McCain foods, which reports no action on salt and provided limited evidence of nutrition policies, stipulates that they “…regard compliance with the law as a minimum standard to be achieved. Our aim is to continuously improve our environmental performance…” [45]. To deliver on this they describe measuring their current impacts, setting environmental targets for improvement and monitoring progress against these targets. This company and several others [46,47,48,49,50,51,52,53] are making contributions to planetary health (or at least report that they are) and this could be coupled with human health in order to increase manufacturer’s action in line with the healthy eating agenda.

## 4. Implications

This study found Australia’s food manufacturers, with few exceptions, do not appear to be making significant “positive (nutrition) changes” to their product portfolios, although half document at least some salt-reduction activities. In light of the increasing rates of hypertension [3] and population salt consumption [4], more could be done.

In terms of the policy ramifications, the Australian Government’s Healthy Food Partnership has the opportunity to endorse the continuation of voluntary targets for food reformulation, set time frames, expand the included products and settings (retail, quick-service, and restaurants) and transparently monitor and report on progress. At this mid-point between the WHO Global Action Plan and the 2025 salt reduction target, there is significant opportunity for Australia to achieve what the UK Responsibility Deal and the Pan-Pacific Sodium Consortium have achieved. Introducing regulatory scaffolding around sodium reduction targets has been suggested as a proactive approach for the Australian government to adopt in order to increase participation in voluntary measures [54].

In the Australian political context, bi-partisan support for food reformulation measures that address public health concerns would be appropriate. With or without bi-partisan commitment, or government support, key stakeholders in the Australian food supply such as food manufacturers, distributors and retailers could proactively respond to international leadership and public health concerns. Some food manufacturing companies are already showing significant innovation and progress.

Similarly, direct engagement of public health organisations with industry on developing and delivering on triple-bottom line policies and priorities has merit. The potential for company directors to bear responsibility for the health effects of company products, such as occurred for producers of tobacco products, lead and asbestos, could benefit from engagement with public health experts.

Manufacturers’ willingness to dedicate sections of their websites, reports and media content to environmental responsibility could provide a platform to support nutrition responsibility too. Significant research on the inter-linked challenges and opportunities for human and planet health via food production and consumption could be used, and relevant stakeholders across the food system could be engaged, to increase the health of the planet and people [42].

### Study Limitations

This study only included publically available information relevant to salt and nutrition and when invited to provide further information, several company’s declined stating it was commercially confidential and many did not reply at all. This means that highly relevant material demonstrating commitment to public health may not have been discoverable. Websites typically include current material and the search may not have located achievements or policies in the past that were relevant in the 2010–2017 period. The content analysis reported themes, however did not objectively assess the validity of claims made by companies. Green washing and corporate irresponsibility by food manufacturers has been identified elsewhere in the literature [42,44,55] and further research could reveal the extent to which this was or is occurring in the Australian food manufacturing sector. The findings should be interpreted with this in mind. Finally, whilst this is only a study of Australian food manufacturers, the method could be easily adopted elsewhere and findings are likely to be applicable considering the high levels of salt consumption from processed foods in contemporary world-wide food supply chains [56].

## 5. Conclusions

This study found Australia’s food manufacturers, with few exceptions, do not appear to be making significant and comprehensive “positive (nutrition) changes” in relation to salt or healthier food products. More could be done to capitalise on current nutrition activities, mobilise manufacturers and support product reformulation to improve the nutrition profile of processed foods. The Healthy Food Partnership is yet to develop a high-level ambitious strategy and implementation plan to improve and accelerate reformulation progress by 2025 and beyond, in response to the significant and rising levels of diet-related diseases in Australia. Comprehensive salt reduction targets and independent monitoring, combined with strong leadership through the Partnership, increased investment and strategic oversight by the Australian government would help manufacturers to reformulate products and reduce population salt intake.

## Figures and Tables

**Table 1:** Proportion of salt intake (%) from food groups using National Health Survey data 2014, adapted from [6]. Reproduced with permission from authors and organization.

Food Group	% Contribution to Overall Dietary Salt (Sodium) for All Persons
Cereal-based products and dishes (all)	24.8
Meat, poultry, game products and dishes (all)	18.3
Cereal and cereal products (all)	18.2
Sauces, dips and condiments (all)	5.9
Soup	4.5
Cheese	3.9

**Table 2:** An evaluation of the FHD reformulation targets, adapted from [25]. Reproduced with permission from authors and organization.

Product Type		Agreed Sodium Reformulation Targets for the FHD	Proportion of Products Not Exceeding Maximum Sodium Target (at Baseline 2009–2012)	Proportion of Products Not Exceeding Maximum Sodium Target (2015)
**Breads**		Max. of 400 mg/100 g	28.0%	86.0%
**Cheese**	Cheddar and cheddar style	Max. of 710 mg/100 g	83.5%	86.4%
	Low moisture mozzarella	Max. of 550 mg/100 g	63.2%	68.4%
	Chilled processed	Max. of 1270 mg/100 mg	37.2%	43.2%
**Processed meats**	Bacon	Max. of 1090 mg/100 g	25.0%	59.0%
	Ham and other cured meats		46.9%	79.7%
	Emulsified luncheon meats	Max. of 830 mg/100 g	22.7%	44.4%
**Potato/Corn/Extruded Snacks (PCES)**	Cereal-based snacks	Max. of 700 mg/100 g	88.4%	92.3%
	Potato chips	Max. of 800 mg/100 g	92.5%	91.8%
	Extruded snacks	Max. of 1250 mg/100 g	95.5%	93.5%
	Salt & vinegar products	Max. of 1100 mg/100 g	52.9%	78.3%
**Ready-to-eat breakfast cereals**		15% reduction across products with sodium levels exceeding 400 mg/100 g	54.5%	83.2%
**Simmer sauces**	Asian style	15% reduction across sauces with sodium levels exceeding 680 mg/100 g	41.0%	59.3%
	Indian style	15% reduction in across sauces with sodium levels exceeding 420 mg/100 g	40.0%	68.0%
	Pasta		33.3%	75.8%
	Simmer (other)		25.0%	45.5%
**Savoury crackers**	Plain crackers (flour-based)	Max. of 850 mg/100 g	76.8%	87.2%
	Flavoured crackers (flour based)	Max. of 1000 mg/100 g	72.3%	78.6%
	Flavoured rice/corn cakes/crackers	Max. of 850 mg/100 g	70.0%	75.7%
**Savoury Pies**	Wet	10% reduction across those with sodium levels exceeding 400 mg/100 g	28.4%	51.2%
	Dry	10% reduction across those with sodium levels exceeding 500 mg/100 g	36.6%	27.6%
**Soups**	Wet/condensed soup products	Max. of 300 mg/100 g	75%	80.0%
	Dry soup products	Max. of 290 mg/100 g	27.2%	77.9%

**Table 3:** Priorities and actions publically reported by Australian food manufacturers—salt.

**Company Name** *Major Relevant Australian-Made Brands*	**Example Relevant Products**	**Documentation of Salt Reduction**	**Example Priorities or Actions**
**Arnotts** *Campbells*	Crackers, savoury biscuits, canned soups	✓	“Campbell Arnott’s …have undertaken a stepwise reduction of sodium in order to meet the Tick’s sodium criteria for soups. In 2004, only 33% of Campbell’s soups met the Tick’s sodium criteria of 300 mg/100 g or less. By 2009, approximately 83% of all Campbell’s soups met the sodium criteria”. Source: Personal communication from Arnotts (28 May 2017)
**Baiada Poultry** *Steggles, Lilydale*	Chicken nuggets, kievs, schnitzels	✓	Steggles supplies products that are compliant with the Australian School Canteen association’s nutrition bodies, which stipulate products must have: “450 mg or less of sodium per 100 g”. Source: “Healthy Chicken for Kids” section of Steggles website (visited 22 May 2017)
**Bega Cheese**	Cheese		
**Bellamy’s Organic**	Toddler snacks, pasta		
**Burra Foods**	Cheese		
**Cerebos** *Asian Home Gourmet, Fountain & Gravox*	Gravies, sauces, ready meals	✓	Fountain has created a healthier range of sauces, which include: “25% less added salt than Fountain regular tomato and barbeque 500 mL squeeze sauces” Source: Fountain sauces website (visited 18 May 2017)
**Coca-Cola Amatil** *SPC, Ardmona*	Baked beans, tinned spaghetti	✓	In 2014 “Reduced salt and sugar in key tomato products helping Australians eat healthier”. Source: 90 year history section of SPC Ardmona website (visited 18 May 2017)
**Cordina Chicken Farms**	Schnitzel, nibbles, burgers		
**Devondale Murray Goulburn**	Cheese, cream cheese, butter		
**Freedom Foods Group** *Freedom Foods*	Cereals, snacks, spreads	✓	“At Freedom Foods…You won’t find any that have more than 600 mg of sodium per 100 g”. Source: “Salt” section of Freedom Foods website (visited 18 May 2017)
**General Mills Holding (Australia)** *Latina Fresh, Nature Valley, Old El Paso, Pasta Master*	Pasta sauce, snacks tacos	✓	Old El Paso offers a “reduced salt taco spice mix”, also “healthy fiesta” burrito kit packet with the Heart Foundation tick. Source: Old El Paso website (visited 18 May 2017)
**George Weston Foods** *Tip Top, Don, KR Castlemaine, Mauri ANZ*	Bread, small goods, bread-making products	✓	“Reducing salt GWF is one of Australia’s first companies to establish a sodium criteria as part of the National Heart Foundation Heart Tick program and Voluntary Sodium Reduction Roundtable initiative. Since 2007, we’ve reduced salt across our breads and small goods, contributing to the removal of more than 340 tonnes of salt from Australian diets.” Source: “Corporate Responsibility at GWF” document, GWF website (visited 11 April 2017)
**Goodman Fielder** *Country Life, Golden Canola, Helgas, Holbrooks, Irvines, La Famiglia Kitchen, Lawsons, Logicol, Meadowlea, MacKenzie, Mighty Soft, Molenberg, Olive Grove, Praise, White Wings, Wonder White*	Bread, pastry, cheese, sauces, spreads, cake mixes	✓	Helgas wraps advertised as salt-reduced wraps: “40% less salt than the market leader”. Source: Helgas website (visited 11 May 2017)
**Green’s Foods** *Waterthins, Poppin, Roccas Deli*	Crackers, popcorn		
**Heinz**	Baked beans, canned tomatoes, sauces	✓	“At Heinz we’re always interested in discovering new way to make our products even more nutritious and appealing—from…to our growing section of reduced sugar and salt products”. Source: Health section, Heinz website (visited 1 May 2017)
**Ingham’s**	Nuggets		
**Kellogg Australia Holdings**	Cereal, snack bars	✓	“2012—We announced that we’d reduced the salt levels in Corn Flakes and Rice Bubbles cereals in Australia by 20%. This reduction meant that since 1997, we’d reduced salt levels across our cereals by up to 59%—that equates to approximately 276 metric tonnes, or more than 4.9 m salt shakers (60 g) removed from Australian diets every year.” Source: Our history section of Kellogg’s website (visited 26 May 2017)
**Mars** *Dolmio, KanTong, Master foods, Uncle Bens*	Sauces, spreads and rice-based ready meals	✓	“We have been progressively reducing salt across our total portfolio in line with our commitment to the Department of Health and Ageing salt reduction targets, and many of our products now carry the National Heart Foundation Tick…” Source: “Food” section of Mars Australia website (visited 17 April 2017)
**McCain Foods**	Ready-meals, pizzas		
**Mondelez Australia** *Vegemite*	Crackers, spread	✓	“Reduced Salt VEGEMITE is best enjoyed by the many Australians consciously reducing their salt intake for health and wellbeing reasons. Older Australians and parents wishing to choose lower salt options for the family will love Reduced Salt VEGEMITE…” Source: Vegemite website (visited 3 April 2017)
**Nestle** *Maggi, Milo, Uncle Toby’s*	Breakfast cereal, bars, drink, noodles, stock	✓	“…The foundation members of the Healthier Australia Commitment…have voluntarily agreed to the following collective targets for reductions … by 2015: Reduce sodium in products by 25 per cent—equivalent to over 270,000 kilograms of sodium removed from the food supply…” Source: Nestle media release 2012, on website (visited 28 April 2017)
**Norco Co-Op**	Butter, cheese		
**Parmalat Australia** *Lemnos, President*	Cheese		
**Patties Foods** *Four’N’Twenty, Herbert Adams, Nanna’s*	Pies, pastries, sausage rolls		
**Pepsico Australia & New Zealand** *Doritos Corn Chips, Nobby’s Nuts, Red Rock Deli, Parker’s Pretzels, Sakata Rice Crackers, Smith’s Chips, Sunbites, Twisties*	Breakfast cereal, snack foods	✓	“The reduction in saturated fat follows Smith’s previous commitment of a 25% reduction in salt content across its product range by 2012. Forty products have thus far been reformulated.” Source: Media release “Smiths—Australia’s favourite chip now has 75% less saturated fat” available on Smiths website (visited 26 April 2017)
**Sanitarium**	Breakfast cereal	✓	Health Star Rating salt standards have set the agenda for their product reformulation work—previously they had their own internal nutrition standards, but the new initiative has superseded this. Source: Personal email (received 30 May 2017)
**San Remo**	Pasta and sauces		
**Scalzo Food Industries**	Snacks		
**Simplot Australia** *Birds Eye, Leggos, Edgell, Lean Cuisine, Harvest, Chiko, I&J, Top Cut, Five Tastes, Simply Great Meals*	Frozen snacks, ready-meals, meal kits, meat, small goods	✓	“We are proud to report that 29 tonnes of salt has been removed from our Leggo’s pasta sauce range as a direct result of [the Food and Health Dialogue]…” Source: Nutrition news section “reducing sodium for better health” dated 10 October 2016 (visited 11 May 2017)
**Sunrice**	Rice, snacks		
**Thomas Foods International**	Burgers, meatballs, sausages		
**Unilever Australia** *Bertoli, Continental, Flora*	Margarine, pasta, sauces, stock, soup	✓	Unilever reports their global progress, but also their progress by nation state. In Australia in 2016 68% of the foods in their portfolio met the salt levels they devised to reach the WHO 5 g per day target. Source: Performance against the USLP global nutrition targets in key countries report (2016)
**Warrnambool Cheese & Butter**	Cheese, butter		

Table Key: Obtained a tick (Reformulation concerning any salt/sodium nutrients; Reduced-salt product lines; Described participation in Food and Health Dialogue, Heart Foundation Tick program, the Healthy Food Partnership, Industry reformulation activities); Did not obtain a tick (Reformulation concerning other nutrients; No evidence of Australian product lines reducing salt/sodium).

**Table 4:** Priorities and actions publically reported by Australian foodmanufacturers—nutrition.

Company Name	Documentation of Nutrition as a Priority/Activity	Example Priorities or Actions
**Arnotts**	✓	“We welcomed the development of the Australian Food and Grocery Council’s (AFGC) Responsible Children’s Marketing Initiative and have pledged its commitment to marketing communications to children under 12 years of age only when it will further the goal of promoting healthy dietary choices and healthy lifestyles in accordance with the core principles set out below…” Source: “Our commitment” section of Arnott’s website (visited 26 April 2017)
**Baiada Poultry**	✓	Steggles supplies products that are compliant with the Australian School Canteen association’s nutrition bodies, which stipulate products must have: “1000 kj of energy or less per 1000 g; 4 g or less of saturated fat per 100 g”. Source: “Healthy Chicken for Kids” section of Steggles website (visited 22 May 2017)
**Bega Cheese**	✓	Manufactures cheese, a nutritious food consistent with the Australian Dietary Guidelines Source: Bega website (visited 7 April 2017)
**Bellamy’s Organic**	✓	Bellamy’s website hosts a blog from Paediatric Dietitian and Nutritionist Susie Burrell. The “Top Five Nutrients Your Toddler Needs” blog entry provides consumers information to increase healthy eating in children Source: Blog 12 September 2016, on Bellamy website (visited 28 February 2017)
**Burra Foods**	✓	Manufactures milk and other dairy products consistent with the Australian Guide to Healthy Eating Source: Burra website (visited 7 April 2017)
**Cerebos**	✓	Fountain “No Added Sugar” Tomato and BBQ Sauces were launched in 2013 and are sweetened using the natural sweetener, Natvia. Source: Personal communication, email (received 18 May 2017)
**Coca-Cola Amatil**	✓	“Why aren’t Australians eating enough legumes? On average Australians eat 18.5 g of legumes, or a quarter of one serve, per week. But this average is deceptive because actually most people are not eating any legumes…” Source: SPC media release 6 August 2012, SPC website (visited 9 April 2017)
**Cordina Chicken Farms**	✓	Manufactures chicken breast, thigh and other minimally processed poultry products consistent with the Australian Guide to Healthy Eating Source: Cordina website (visited 7 April 2017)
**Devondale Murray Goulburn**	✓	Infographic on the benefits of dairy products Source: Dairy goodness section, Murray Goulburn website (visited 19 May 2017)
**Freedom Foods Group**	✓	Provides consumers with information on allergens and food composition in their products. In addition, sections on body mass index and weight management, nutrition and cardiovascular health and mental health. Source: “Your Health and Wellbeing” section, Freedom Foods website (visited 15 April 2017)
**General Mills Holding (Australia)**	✓	“Australia: Compliance with the Responsible Child Marketing Initiatives of the Australian Food and Grocery Council” Source: General Mills Global Responsibility 2017 report, p. 28
**George Weston Foods**	✓	“Helping consumers make healthy choices: GWF is a member of the GoScan program, which helps consumers access trusted product information from their mobile phone”. Source: “Our quality promise”, Corporate responsibility at GWF, website (visited 11 April 2017)
**Goodman Fielder**	✓	Wonder White bread products displayed online with information on their composition and associated nutrition claims. Source: “Health and Nutrition”, Wonder White website (visited 18 April 2017)
**Green’s Foods**	✓	Removed trans-fats in popcorn “Poppin” products Source: Personal communication (11 April 2017)
**Heinz**	✓	Heinz infant feeding advisory service. Source: Heinz for baby website (visited 4 May 2017)
**Ingham’s**	✓	Manufactures chicken breast, thigh and other minimally processed poultry products consistent with the Australian Guide to Healthy Eating Source: Ingham website (visited 7 April 2017)
**Kellogg Australia Holdings**	✓	Kellogg’s one of the first manufacturers to employ dietician and continues to employ and utilise this skill set. Source: Nutrition at its best, Kellogg’s website (26 May 2017)
**Mars**	✓	“To help our consumers make informed choices, we’ve renovated our products and introduced more nutritional information. Now it’s easier than ever to be aware and compare”. Source: Mars “Making Chocolate Better Program”, Mars chocolate website (11 April 2017)
**McCain Foods**	✓	“Healthy Choice” prepared meals product line Source: McCains website (visited 29 March 2017)
**Mondelez Australia**	✓	The “Call for Wellbeing” report includes their targets for increasing whole grain content, reducing portion sizes, reducing saturated fat content and displaying front of pack labelling, and progress against these targets Source: “The Call for Well-being” 2015 Progress report, available on Mondelez website (visited 11 April 2017)
**Nestle**	✓	Development of the “together counts” website to “…educate the community about the concept of energy balance, promoting healthy eating and physical activity...” Source: Media release “Food industry commits to reduce salt, saturated fat and energy”, 10 October 2012
**Norco Co-Op**	✓	Manufactures milk, a nutritious food consistent with the Australian Dietary Guidelines Source: Norco website (visited 7 April 2017)
**Parmalat Australia**	✓	Manufactures milk, a nutritious food consistent with the Australian Dietary Guidelines Source: Parmalat website (visited 7 April 2017)
**Patties Foods**	✓	Patties pies display the voluntary front of pack labelling: the Health Star Rating system Source: Patties website (19 April 2017)
**Pepsico Australia & New Zealand**	✓	“With new nutritional goals informed by the latest guidelines from the World Health Organization and others, we plan to further reduce added sugar, sodium and saturated fat levels, while growing our “Everyday Nutrition” brands faster than the balance of our portfolio”. Source: Pepsico Annual report 2016, p. 7
**Sanitarium**	✓	Manufactures breakfast cereals consistent with the Australian Dietary Guidelines Source: Sanitarium website (visited 7 May 2017)
**San Remo**	✓	Introduction of new product line—pulse pasta. “Pulse Pasta is 100% Lentils, Peas, Borlotti Beans and Chickpeas. Pulses are a good source of protein to keep you fuller for longer, rich in soluble fibre for digestion and some are also a great source of iron for plenty of energy to fuel your body…it is also Gluten Free and Vegan friendly and you can use it just like normal pasta!” Source: San Remo website (visited 24 May 2017)
**Scalzo Food Industries**	✓	Manufactures nut-products, consistent with the Australian Dietary Guidelines Source: Scalzo website (visited 17 May 2017)
**Simplot Australia**	✓	“Simplot has taken the initiative to include the HSR icon on all Simplot branded food products available via retail outlets at your local supermarket”. Source: Nutrition commitment section Simplot website (visited 25 May 2017)
**Sunrice**	✓	Consumer information on nutritional benefits of rice, dietary recommendations regarding serves of cereals and information on low-glycemic index foods Source: Sunrice website (visited 7 May 2017)
**Thomas Foods International**	✓	Manufactures meat products consistent with the Australian Dietary Guidelines Source: Thomas Foods website (visited 7 May 2017)
**Unilever Australia**	✓	Unilever nutrition targets Source: Performance against the USLP global nutrition targets in key countries report 2016.
**Warrnambool Cheese & Butter**	✓	Manufactures cheese, a nutritious food consistent with the Australian Dietary Guidelines Source: WCB website (visited 7 April 2017)

Table Key: Obtained a tick (Reformulation concerning nutrients, not including sodium, such as fat, sugar, kilojoules, protein, fibre, vitamins, minerals; Development of healthier-product lines; Providing consumer nutrition information, including participation in the Health Star Rating; Producing a core food “healthy” product consistent with the Australian Dietary Guidelines; Marketing standards (i.e., advertising to children)); Did not obtain a tick (Food safety, quality assurance and allergen compliance; Reformulation concerning non-nutrient components (i.e., artificial colours/flavours, organic health claims); Manufactured only discretionary foods).
